# A study on diameter-dependent support selection of the tendrils of *Cayratia japonica*

**DOI:** 10.1038/s41598-022-08314-w

**Published:** 2022-03-15

**Authors:** Kazuya Saito

**Affiliations:** Faculty of Design, Kyushu University, Fukuoka, 815-8540 Japan

**Keywords:** Plant sciences, Plant ecology

## Abstract

Organisms make decisions when they perceive cues of varying intensities. In case of climbing plants, the diameter of supports in contact (tree or stem) is an important cue for their growth as plants that coil around a support with large diameter are unable to maintain tensional forces required for continued attachment to the support. The negative association between the diameter and the climbing success has been reported since Darwin published his study on climbing plants. However, it is not known if a climbing plant makes a decision to avoid a support with larger diameter. Here, we tested this possibility by observing the coiling response of tendrils of *Cayratia japonica* to supports with different diameters. The coiling success of the tendrils was affected by the diameter of the support and the tendril lengths. We propose a decision tree to describe the different phases of the coiling response and demonstrated that the tendrils change their coiling shape depending on the support diameter and the tendril length. To understand the behavioural rules regulating the phase pattern, we constructed a simple model with two assumptions on the tendril movement, (1) when the tendrils receive a contact stimulus, they begin to coil from around the contact point and (2) there is a minimum coiling angle at which the tendrils coil up, once the tendril starts coiling. Image analysis and 3D motion tracking technique revealed that the movement of the tendrils were consistent with the two assumptions of the model. The results suggested that the tendrils flexibly changed the coiling shapes depending on the support diameter and simple behavioural rules could regulate this diameter-dependent response.

## Introduction

The behaviour of an organism is defined as its phenotypic response to the perceived cues of an event or change in its environment in the course of its lifetime, which is relatively rapid and generally reversible^1^. When there is a variation in the value of the perceived cues, organisms tend to select an option from a set of alternative responses based on the value of the cues. In animals, these responses have been regarded as decision making behaviour and play an essential role in survival, foraging, and reproduction^2–4^. Recently, growing evidences suggest that plants could also perceive cues with different values and change their phenotypes plastically according to the values of the cue and their own conditions^5–8^. In this sense, plants can be regarded as being capable of making decisions^9–12^. However, plants do not make decisions using the same behavioural and physiological mechanisms as the animals do because the plants have no nervous system and brain. Rather, plants mostly have autonomous modular units, that are generally less well-developed than animals^1,13^. Therefore, it is expected that behavioural and physiological mechanisms underlying the plants’ decision-making are largely caused by non-integrated local reactions^13,14^.

Climbing plants can be a model system to study the decision-making in plants because they show rapid movement in response to environmental cues^15–18^. For climbing plants, finding a suitable host plant on which they can climb is one of the most important factors affecting their growth and development^19^. They can perceive several cues from the host plants (e.g. darkness and host volatiles) to locate the host plants quickly^20–22^. However, there is a large variation in the values of the host plant as a coiling support. For example, when support diameter increases beyond a certain point, climbing plants are unable to maintain tensional forces that facilitate coiling and attachment to the support^19,23–25^. Thus, a support with a large diameter is not suitable for coiling and climbing. In fact, negative associations have been reported between the support (tree) diameter and colonisation success in the field^23,25–27^ with Darwin being the first one to report it^15^. However, it is not known whether climbing plants determine to avoid an unsuitable host by changing their coiling response depending on the quality of the support. This is a central question in the behaviour of climbing plants^18^.

Tendrils of climbing plants are specific organs modified from leaves, leaflets, shoots, and stipules and appear to have evolved independently in many families^28,29^. Compared to twining climbers, tendril-bearing plants have a narrower response range. Thus, the upper limit of the support diameter that tendrils can coil successfully is relatively small^18^. Maintaining attachment and coiling around a support that is too large in diameter results in loss of time and energy. For tendril bearing climbing plants, it would be important to avoid hosts with large diameters. It has been observed that the tendrils grasp onto supports by methods other than coiling in cases where they come in contact with thick non-cylindrical structures, such as walls and corners (Fig. S1). A typical example is the clip shape coiling of *C. japonica*, which will be discussed later. The mechanism by which a climbing plant selects the ideal tendril shape for gripping an object of unknown shape needs to be elucidated. This problem is also interesting from the perspective of biomimetic engineering^30–33^. Grabbing objects is the most basic function of robots used in manufacturing and logistics. They are systems that specialize in the shape and size of the objects to be grabbed (for example, screws) and are usually equipped with advanced sensors that determines the shape and posture of the targets. It is an interesting engineering challenge to properly grasp an object of unknown shape using limited information.

To test the ability to make decisions in host plant selection by climbing plants, we focused on *Cayratia japonica*, a perennial vine of Vitaceae family. Previously, it was reported that the tendrils of *C. japonica* have the ability to discriminate and avoid unsuitable hosts, their own and conspecific leaves, by detecting the chemical cues from conspecifics^34,35^. In this study, we investigated the ability of *C. japonica* to detect the physical cues. The main research question is: Are the tendrils of *C. japonica* able to detect the support diameter and thus avoid unsuitable host plants? This decision is not an animal-like active decision to take another option, but a passive and automatic mechanism that makes it easier to select a support with an appropriate diameter. Firstly, we examined whether the support diameter and the tendril length affect the coiling success. Secondly, to determine the change tendrils change their coiling response depending on the support diameter and tendril length, we described the phase pattern of coiling responses based on the observations. Thirdly, we constructed a simple verbal model to explain the observed phase patterns of the coiling response depending on the support diameter. Finally, we experimentally verified a key rule for the coiling movement assumed in the model.

## Materials and methods

### Study species

*Cayratia japonica* (Thunb.) Gagnep. (also known by its synonym *Causonis japonica* and the common names bushkiller, and yabu garashi) belongs to the Vitaceae family. It is a perennial herbaceous vine, which is widespread in Japan and is not a protected plant or endangered species. In October 2016, we collected 40 *C. japonica* triploid rhizomes from the Institute of Sustainable Agro-ecosystem Services (ISAS), University of Tokyo. The rhizomes were cut into segments, 30 to 40 cm long, and transplanted individually into pots containing a commercial vermiculite product (‘Golden’ vermiculite; Iris Ohyama Co. Ltd., Sendai, Japan). These individuals were grown under heated greenhouse conditions. The plants were watered sufficiently. Plants used for the experiment were at least 20 cm in height, with at least one tendril. Each tendril was used only once. All experiments were carried out when the room temperature was between 20 and 30 °C.

Experimental research and field studies on plants in this work comply with the IUCN Policy Statement on Research Involving Species at Risk of Extinction and the Convention on the Trade in Endangered Species of Wild Fauna and Flora. All plant materials involved in the study were collect with the permission of the University of Tokyo.

### Observation of coiling pattern

To examine the effects of support diameter on coiling pattern and the final coiling success of tendrils of *C. japonica*, we observed the temporal changes in the coiling response and the coiling success in response to supports with various diameters (φ = 10, 12, 15, 18, 20, 24, 30, 32, and 35 mm). The experiment began on 1 November and concluded on 2 December 2016. The tendrils were provided with one of the supports of different diameters. The contact point was in the middle of tendril. The support was placed at right angle with respect to the direction of extension of the tendril. After the contact, we recorded the type of initial movement because we noticed that the tendrils in contact with the support showed two distinct coiling responses initially (within 5 min after the start of coiling). Then, the temporal changes in coiling shape, the final coiling success until 3 h after starting the experiments, and the time to detach were recorded. Finally, phase patterns for the temporal changes in coiling responses of the tested tendrils were described. The sample size of the experiment was 87. The generalized linear model analysis was used to examine the effects of tendril length and support diameter on the coiling success and the phase pattern changing of tendrils.

### Model proposal

Based on the observation of the coiling response, we provided a simple verbal model describing how the phase of initial response in the first phase of coiling occurs because the initial phase plays a critical role in the overall phase pattern of the coiling response.

To verify the proposed model, we tested (1) whether the tendril begins to coil from around the contact point and (2) whether the tendril in contact with the support has minimum coiling angle by image analysis. We provided a bamboo support to the tendril at right angle for 60 s and then detached it. To eliminate the effect of the circumnutation of the plant stem, the plants were fixed to bamboo sticks with plastic ties^34^. This fixation limits the spatial range of the tendril to such an extent that only the apical areas of the tendril can have contact with the support. The lower parts of the sticks were inserted into clay. We took 12 pictures every 60 s after the detachment, using a camera located immediately above the tendril. The coiling angle of the tendril in each time frame was measured using ImageJ software (NIMH, National Institute of Mental Health, Bethesda, Maryland, USA). The sample size used in the experiment was 10.

### 3D motion tracking

To analyse the initial coiling motion three dimensionally, we used an image-based 3D motion tracking system using a multi-camera. The tendrils were marked with a fine point marker at 5 mm intervals. To eliminate the effect of curling in the tendril that occurs during marking, the experiment was conducted after one hour when the tendril returned to a straight line and became stable. The coiling movement was observed when in contact with the rods of diameters 1 mm and 40 mm. The images for analysis were acquired from different directions by synchronous shooting at 1-min intervals with three cameras (Nikon D5500, Nikon Corporation, Japan). A 3D motion tracking software (DIPP-Motion V/3D, DITECT Co. Ltd., Japan) was used to calculate the 3D coordinates of each marker.

## Results

### Effects of support diameter and tendril length on coiling success

**Figure 1:**
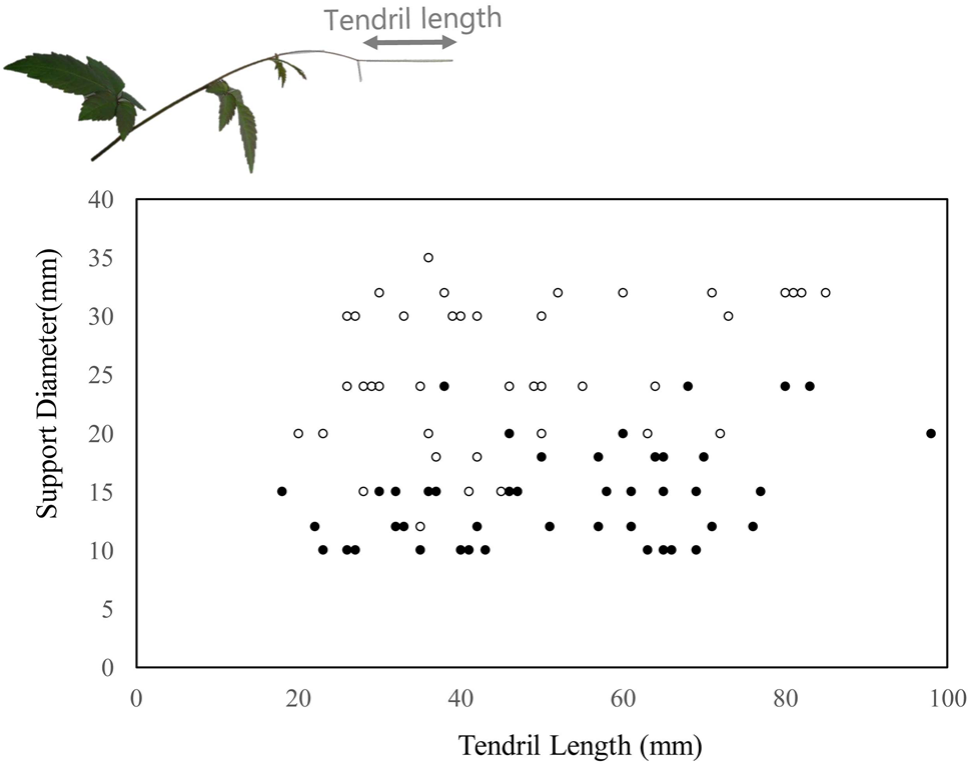
Relationship between tendril length, support diameter and coiling success. Each dot represents the coiling success (black) or failure (white).

We found that both the support diameter and the tendril length affected coiling success (Table S1). The probability of coiling until three hours after the experiment was lower when the diameter of the support was larger and increased as the tendril length increased (Fig. 1).

### Phase pattern of coiling response

**Figure 2:**
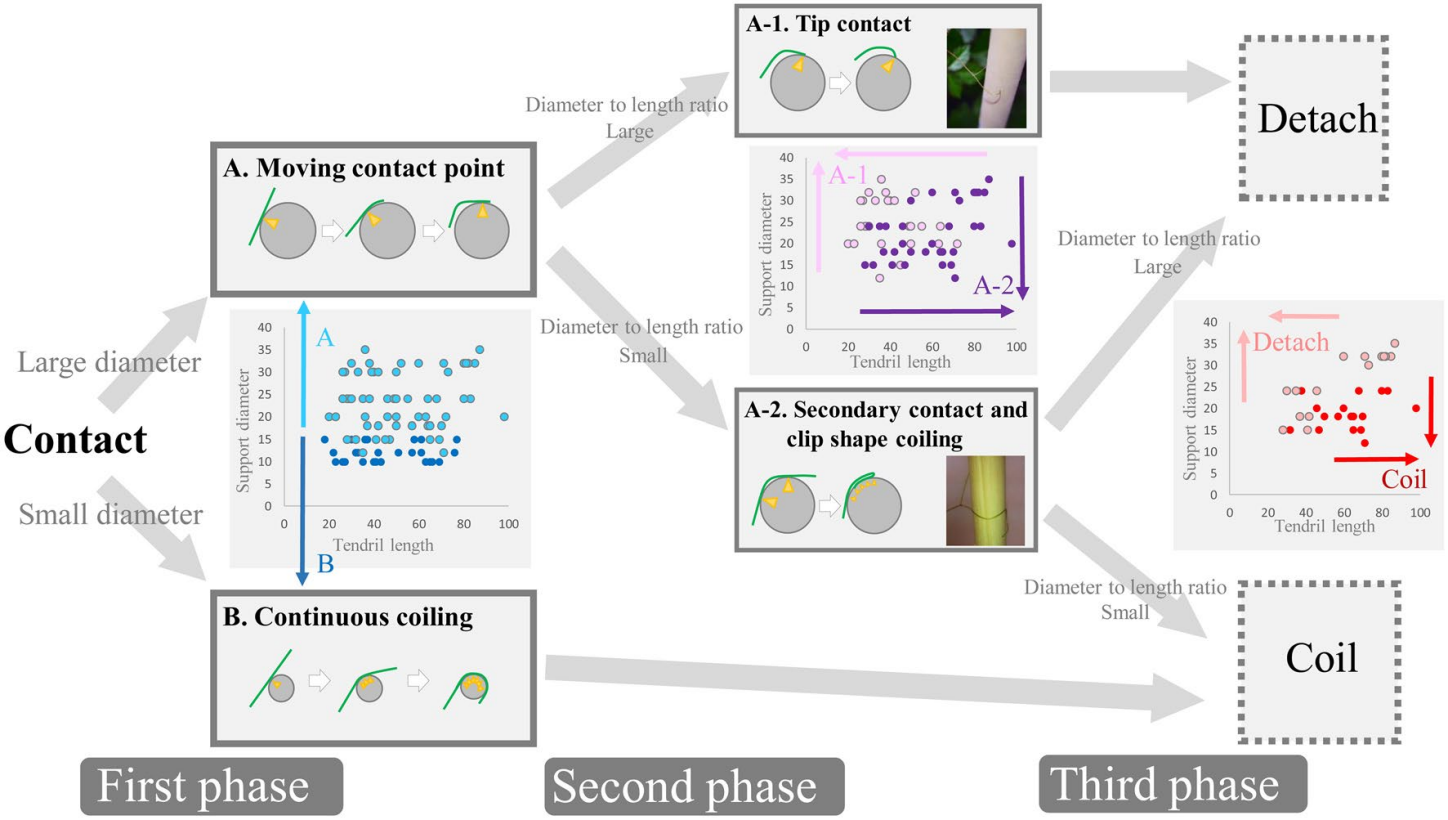
Phase pattern of coiling response. Temporal change in coiling response with three steps was obtained from direct observation. The illustrations and pictures in the frame represent typical examples of the coiling form in each step. For the illustration, green line represents the shape of the tendrils and yellow triangles represents the contact points between the tendrils and the supports. Three scatter plots represent the effects of tendril length and support diameter on the results of the phase pattern. The left, centre, and right plots represent the results of first, second, and third phases, respectively.

Based on the observation of the coiling response to supports with different diameters, we describe the phase pattern of coiling response with three steps (Fig. 2). In the first step of the phase, the tendrils in contact with the support showed two distinct coiling responses in the initial movement (within 5 min after the coiling start). One of the initial coiling responses was defined as ‘continuous coiling’ where the tendrils coil normally while being in continuous contact with the support and without leaving the initial contact point (‘continuous coiling’ in Fig. 2, Supporting Video 1). The other form of initial coiling was defined as ‘moving contact point’ where the tendril coils by leaving the initial contact point and moving the contact point to the tip of the tendril (‘moving contact point’ in Fig. 2, Supporting Video 2).

While all the tendril that showed ‘continuous coiling’ around the support until the end of the experiment, tendril that showed ‘moving contact point’ was diverged into two coiling forms in the second step (Fig. 2). One of the shapes of coiling was defined as ‘tip contact’ where the contact point of the tendril reached to the tip and the tendrils are in contact with the support only at the tip (‘tip contact’ in Fig. 2, Supporting Video 3). All tendrils that showed ‘tip contact’ were detached from the support until the end of the experiment. Another shape was defined as ‘clip shape coiling’ where the tendrils curled in the middle of coiling and attained the shape of a clip (‘clip shape coiling’ in Fig. 2, Supporting Video 2). The ‘clip shape coiling’ seemed to be formed when the tendrils in ‘moving contact point’ make secondary contact with the support at the root of the tendrils and begin to coil again from the secondary contact point. In the third step of the phase, some of the tendril of ‘clip shape coiling’ succeeded in coiling while the others detached. The time taken to detach was 18 ± 12.20 min (mean ± SD) for ‘tip contact’ and 63 ± 28.31 min (mean ± SD) for ‘clip shape coiling’.

### The factors that cause phase pattern

In the first step of the phase pattern, the support diameter had a significant effect on the formation of phase pattern (Table S2). The tendrils showed ‘continuous coiling’ when the support diameter was smaller and ‘moving contact point’ when the support diameter was larger. The bifurcation point was around 10–15 cm in diameter (left figure in Fig. 2). There was no effect on tendril length in the first step of the phase (Table S2).

In the second step of the phase pattern, both the support diameter and the tendril length had significant effects on phase patterns (Table S2). The tendrils tended to show ‘tip contact’ when the support diameter was larger and the tendril length was shorter. On the other hand, the tendrils showed ‘clip shape coiling’ when the support diameter was smaller and the tendril length was longer (central figure in Fig. 2).

In the third step of the phase, both the support diameter and the tendril length had significant effects on the formation of the phase pattern (Table S2). The probability of coiling until three hours after the experiment was lower when the diameter of the support was larger and the tendril length was shorter (right figure in Fig. 2).

### Proposed model

**Figure 3:**
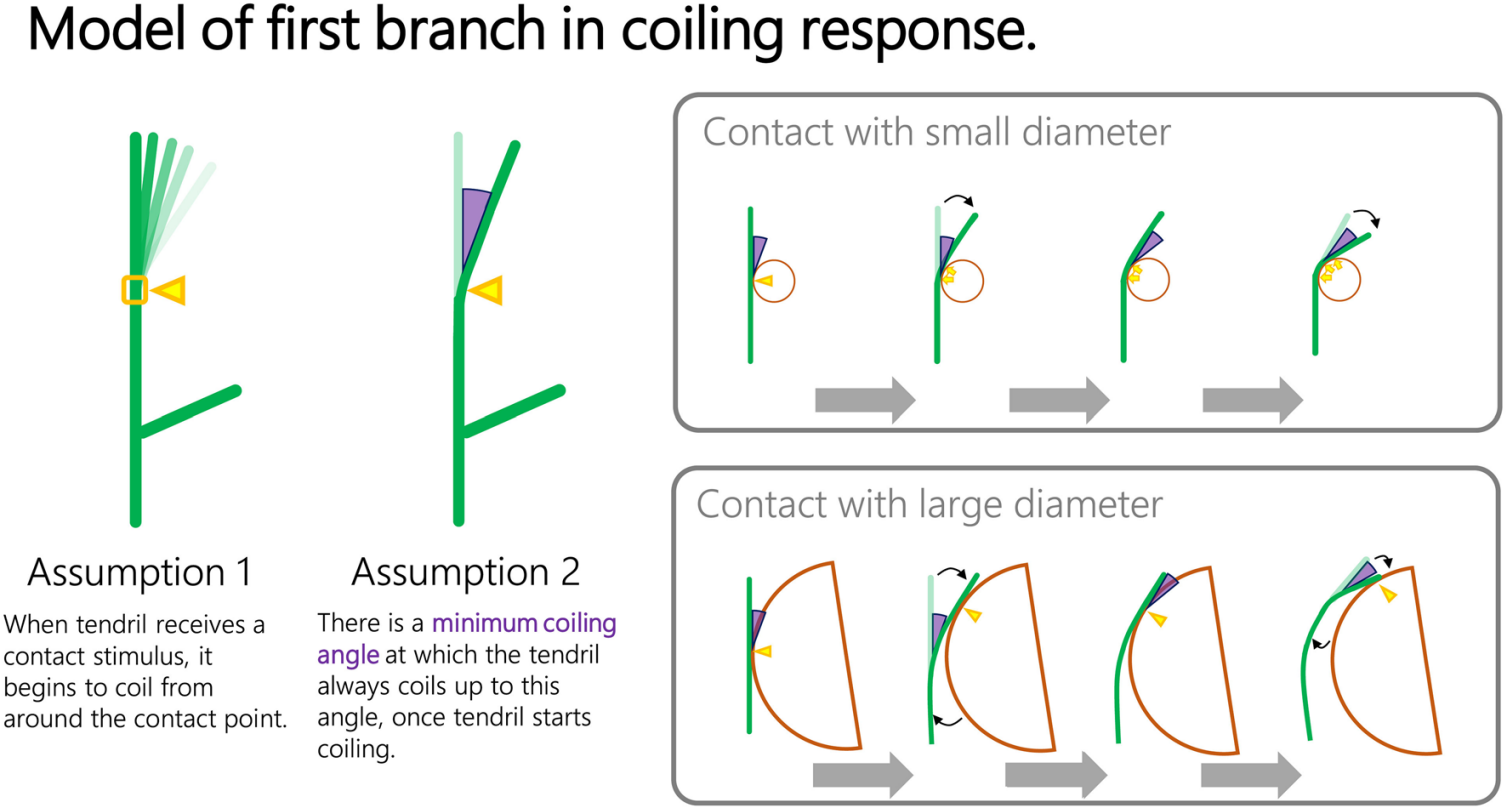
Model that causes the initial phase of coiling response. Two illustrations on the left represents the rules of coiling movement assumed in the model. The green line represents the shape of the tendrils and the yellow triangles represents the contact points between the tendrils and the supports. The purple triangle means the minimum coiling angle of the tendrils. Top and bottom right figures illustrate the expected coiling response when the tendrils are in contact with a support of small and large diameter, respectively.

In the first phase pattern of the coiling response, the tendril showed two distinct initial movements (‘continuous coiling’ and ‘moving contact point’) depending on the support diameter. The initial phase plays a critical role in avoiding supports with large diameter (Fig. 2). Therefore, we constructed a simple verbal model to explain the mechanism regulating the first phase, assuming two kinds of behavioural rules of coiling (Fig. 3). The first assumption is that when the tendrils received a contact stimulus, and began to coil around the contact point. The second assumption is that there is a minimum coiling angle at which the tendrils always coil up once they start coiling.

Under these assumptions, the coiling response will phase depending on the support diameter (and surface curvature) of the contacted support. If the tendrils encounter a support with small diameter having a sufficiently large curvature to achieve the minimum coiling angle, the tendrils will coil around the support while being in continuous contact with the support and without leaving the initial contact point (right upper in Fig. 3). On the other hand, if the tendrils come in contact with a support of large diameter having small curvature, the tendrils get away from the initial contact point to achieve the minimum coiling angle and contacts the support at another point closer to the tip of the tendril. Then, the new contact point becomes a new stimulus for coiling, and the tendrils start coiling again to achieve the minimum coiling angle from around the new contact point. By repeating this response, the contact point will move to the tip of the tendril (‘moving contact point’, right bottom in Fig. 3).

**Figure 4:**
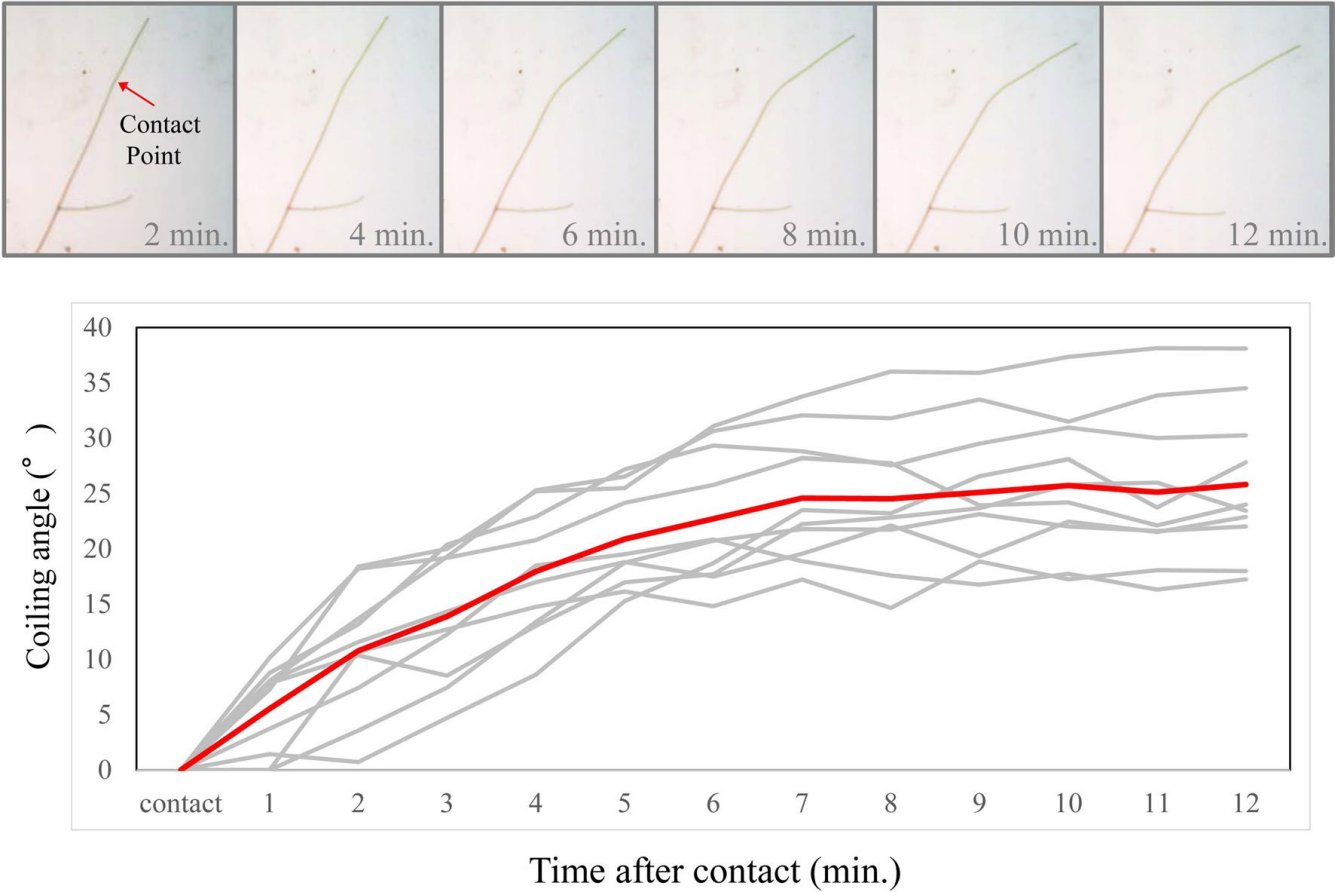
Change in coiling angle after contact. (**Top**) A representative picture of temporal change in coiling angle. (**Bottom**) Temporal changes in coiling angle. Gray lines represent the change in coiling angle of each tendril (n = 10) and red line represent the average.

**Figure 5:**
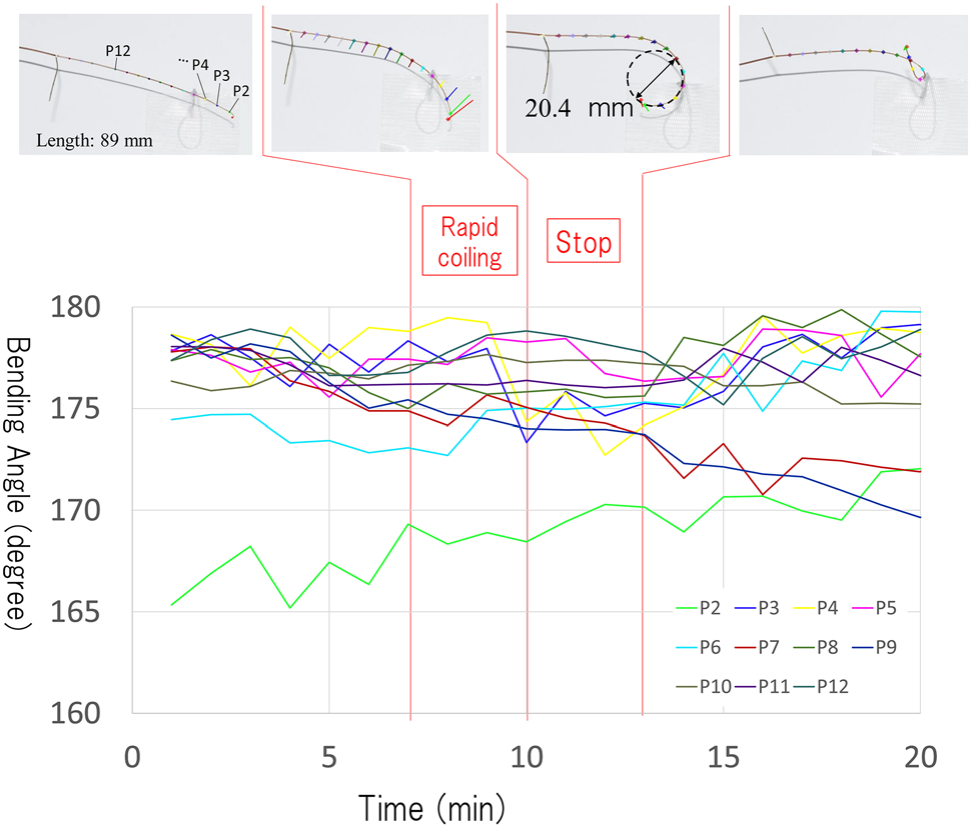
3D motion tracking of the tendril coiling on thin support. Tendril of 89 mm length was placed in contact with 1 mm diameter rod. The vertical axis of the graph is the bending angle at each marked position. After the region where the angle changes rapidly (transform stage), the region with almost no change (stop stage) appears. The representative shape in each stage is shown in the upper figures. The coiling diameter in stop stage that was calculated from the 3D coordinates of the markers was about 20.4 mm.

**Figure 6:**
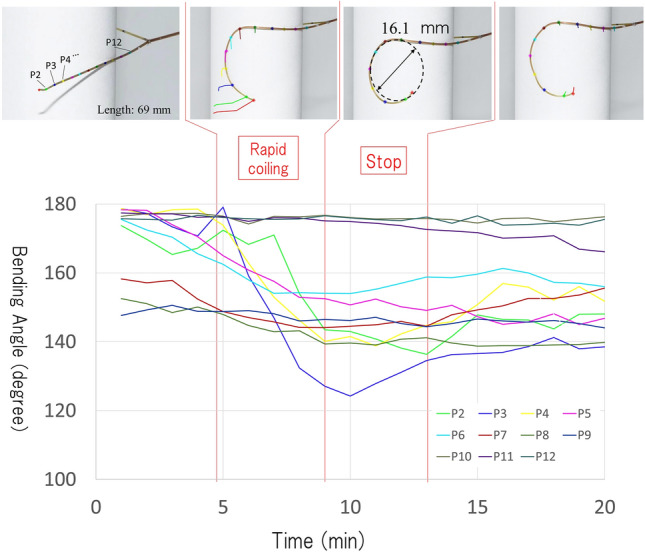
3D motion tracking of the tendril coiling on thick support. Tendril of 69 mm length was placed in contact with 40 mm diameter rod. As in Fig. 5, after the region where the angle changes rapidly (transform stage), the region with almost no change (stop stage) appears. The coiling diameter in stop stage that was calculated from the 3D coordinates of the markers was about 16.1 mm.

Image analysis showed that the tendril of *C. japonica* began to coil from around the contact point at the minimum coiling angle, as assumed in the model. The tendrils that contacted the support for 60 s started to coil at a certain degree from around the contact point (upper pictures in Fig. 4) even after detaching from the support. The coiling angle increased immediately after detaching and continued until it reached 25° on average (bottom in Fig. 4). The results of 3D motion tracking support our hypothesis. Figures 5 and 6 show the coiling motions of the tendrils when in contact with rods of diameters 1 mm and 40 mm, respectively. Each line indicates the position of the marker on the tendril. The vertical axis of the graph is the bending angle calculated from the positions of the front and back points (e.g., the bending angle on point P_4_ is calculated as ∠P_3_ P_4_ P_5_). 180 (°) of the bending angle indicates straight line. In both graphs, there is a region where the angle changes rapidly after contact (rapid coiling stage), followed by a region where the angle hardly changes (stop stage). The actual movements can be confirmed in Video 4 and 5. The coiling diameters in the stop area, calculated from the 3D coordinates of the markers, were about 20.4 and 16.1 mm.

## Discussion

Our results showed that the tendrils of *C. japonica* tend to coil at a fixed diameter until contact occurs regardless of the shape and size of the object. The experimental results and observations revealed that this concept has three advantages. One is ensure grasping by coiling to a small diameter immediately after contact. If the diameter of the contacted support is 15 mm or less, tendrils will successfully grip with higher probability. Since the circumnutation of the plant stem does not stop even during coiling, the support will be far away if the tendril spends a lot of time on gripping.

The second effect is to avoid improper thick support around which it cannot coil. The ability to change the coiling response depending on the support diameter may have an adaptive significance in the host plant selection of *C. japonica*. Especially, the coiling form of ‘tip contact’ was induced with a combination of a small tendril length and a large support diameter, this seemed to contribute to quick detachment from the support (the average time to detach was 18 min). This response mashory reduce the wastage of time and energy spent on trying to coil around unsuitable tree or stem that has much larger diameter. In addition, this response might have an advantage of quickly detaching the tendrils from unsuitable objects that have small surface curvature, such as large stones and leaves with slippery surfaces.

The third effect is the preparation for phase into a clip-shaped gripping pattern. To discuss the advantages of clip shape coiling, it is necessary to consider that the natural supporting structures are unlike the smooth cylindrical supports used in the experiment, and they usually have many protrusions on their surface. It is easy to imagine that a small circle generated by the initial coiling (clip tip) acts like a hook and gets caught in the protrusion of the structure.

In the second phase pattern of coiling, the tendrils of ‘moving contact point’ was diverged into two coiling shapes (‘tip contact’ and ‘clip shape coiling’) depending on both the support diameter and the tendril length. Based on direct observation, it seems that this phase was affected by the presence or absence of secondary contact between the tendril and the support. If the tendrils did not make the secondary contact, the tendrils of ‘moving contact point’ became ‘tip contact’ and detached from the support. If the tendrils made a secondary contact with the support, it started secondary coiling from the point of secondary contact and became ‘clip shape coiling’. The relatively long tendrils tend to make the secondary contact with the support, as result, these longer tendrils showed ‘clip shape coiling’. However, the variance that could not be explained by both the support diameter and the tendril length (central figure in Fig. 2) suggested that other factors also affect the second phase. For example, the microscopic motion of the tendrils due to the rotatory movement of the entire plant will affect the likelihood of the secondary contact and consequently, affects the selection between ‘tip contact’ and ‘clip shape coiling’. The rotatory movement of the entire plant will also affect the third phase of the tendril coiling response (coiling success or failure of the tendrils of clip shape coiling). From the conclusions, the following answer regarding the above-mentioned scientific question can be obtained. The tendril does not have the function of directly detecting the diameter of the support, but the phase pattern changing of the coiling operation makes it possible to automatically avoid the support with a large diameter.

Although the diameter-dependent support selection as revealed in this study is an important factor, some unsolved problems remain in the climbing mechanism. For example, the role of the short arm of the tendril is still unclear. It is easy to imagine that the short arm wraps around the support from the other side of the long arm to create a loop, making grip easier. However, it is unclear how the contact stimulus to the short arm affects the phase pattern of coiling. It is expected that new findings will be obtained by increasing the variation of experiments such as the shape and contact position of the support. Removing the short arm may also be an effective method to test its role.

Coiling of tendrils in many climbing plants, including those in the Vitaceae family, is thought to be caused by contraction of the gelatinous fibres (G fibres) in the tendrils after contact stimuli^36–38^. In the Vitaceae family, tendrils have a cylinder of cortical G fibres and thus can coil in many different directions^36^. Considering the physiological mechanism of coiling, the minimum coiling angle found in *C. japonica* might be caused by the constant contraction rate of G fibres on the contact side of the tendrils. Future research needs to address the exact physiological mechanism of the minimum coiling angle. Karban^13^ suggested that the physiological mechanisms underlying behavioural responses in plants tend to be caused by non-integrated, local reactions. Our results implied that this is true for decision-making in the tendrils of *C. japonica* because the first phase of the coiling response can be explained by the two behavioural rules inherent in the tendrils. Changes in the coiling responses depending on the support diameter may be caused by local reactions in the tendril.

In conclusion, we reported a new type of decision making in tendrils of *C. japonica* that can change the coiling forms depending on the diameter of the support in contact and their own length, in addition to avoiding supports with a relatively large diameter than itself. Our results suggest that the phase pattern in the tendril coiling response depending on the support diameter can be explained by a simple model assuming two rules for the coiling movement. From our (organisms with a nervous system) point of view, it seems to require a relatively complex information processing system to evaluate and respond to the diameter of contacted objects. In contrast, the present study implied that the coiling response depending on the support diameter of the tendrils might be achieved by a simple local reaction. Plants might exhibit complex behaviours with simple behavioural and physiological mechanisms based on a few local rules. These results show the possibility of creating a new plant-type robot arm that can grasp objects of various shapes using an autonomous decentralised unit. Arms that can pick a variety of shapes without the use of sensors or processors have a great engineering potential to simplify pickup systems in manufacturing and logistics industry.

## Supplementary Information

Supplementary Information 1.

Supplementary Video 1.

Supplementary Video 2.

Supplementary Video 3.

Supplementary Video 4.

Supplementary Video 5.

## References

[1] Silvertown J, Gordon DM (1989). A framework for plant behavior. Annu. Rev. Ecol. Syst..

[2] McFarland DJ (1977). Decision making in animals. Nature.

[3] Stephens DW (2008). Decision ecology: Foraging and the ecology of animal decision making. Cogn. Affect. Behav. Neurosci..

[4] Davies NB, Krebs JR, West SA (2012). An Introduction to Behavioural Ecology.

[5] Cahill JF (2010). Plants integrate information about nutrients and neighbors. Science 80.

[6] Cahill JF, McNickle GG (2011). The behavioral ecology of nutrient foraging by plants. Annu. Rev. Ecol. Evol. Syst..

[7] Meyer K, Soldaat L, Auge H, Thulke H (2014). Adaptive and selective seed abortion reveals complex conditional decision making. Plants Am. Nat..

[8] Simon FW, Hodson CN, Roitberg BD (2016). State dependence, personality, and plants: Light-foraging decisions in *Mimosa pudica* (L.). Ecol. Evol..

[9] Trewavas A (2009). What is plant behaviour?. Plant Cell Environ..

[10] Trewavas A (2005). Green plants as intelligent organisms. Trends Plant Sci..

[11] Reid CR, Garnier S, Beekman M, Latty T (2015). Information integration and multiattribute decision making in non-neuronal organisms. Anim. Behav..

[12] Trewavas, A. J. Plant behavioural ecology. In *eLS*. 1–9 (Wiley, 2007).

[13] Karban R (2008). Plant behaviour and communication. Ecol. Lett..

[14] Trewavas A (2005). Plant intelligence. Naturwissenschaften.

[15] Darwin C (1875). On the movements and habits of climbing plants. J. Linn. Soc. Lond..

[16] Darwin, C. *The power of Movement in Plants* (John Murray, 1880).

[17] Atala C, Gianoli E (2008). Induced twining in Convolvulaceae climbing plants in response to leaf damage. Botany.

[18] Gianoli E (2015). The behavioural ecology of climbing plants. AoB Plants.

[19] Gianoli E, González-Teuber M (2005). Effect of support availibility, mother plant genotype and maternal support environment on the twining vine Ipomoea purpurea. Plant Ecol..

[20] Strong D, Ray T (1975). Host tree location behavior of a tropical vine (*Monstera gigantea*) by skototropism. Science.

[21] Runyon J, Mescher M, De Moraes C (2006). Volatile chemical cues guide host location and host selection by parasitic plants. Science.

[22] Guerra S (2019). Flexible control of movement in plants. Sci. Rep..

[23] Putz FE (1984). The Natural History of Lianas on Barro Colorado Island, Panama. Ecology.

[24] Goriely A, Neukirch S (2006). Mechanics of climbing and attachment in twining plants. Phys. Rev. Lett..

[25] Carrasco-Urra F, Gianoli E (2009). Abundance of climbing plants in a southern temperate rain forest: Host tree characteristics or light availability?. J. Veg. Sci..

[26] Putz FE, Chai P (1987). Ecological studies of lianas in Lambir National Park, Sarawak, Malaysia. J. Ecol..

[27] Nabe-Nielsen J (2001). Diversity and distribution of lianas in a neotropical National Park, neotropical rain forest, Yasuní Ecuador. J. Trop. Ecol..

[28] Putz F, Mooney H (1991). The Biology of Vines.

[29] Isnard S, Silk W (2009). Moving with climbing plants from Charles Darwin’s time into the 21st century. Am. J. Bot..

[30] Walker, I. D. Biologically inspired vine-like and tendril-like robots*. *In *IEEE 2015 science and information conference (SAI)* 714–720 (2015).

[31] Bastola AK (2021). Cactus-inspired design principles for soft robotics based on 3D printed hydrogel-elastomer systems. Mater. Des..

[32] Wooten MB, Walker ID (2018). Vine-inspired continuum tendril robots and circumnutations. Robotics.

[33] Fiorello I, Del Dottore E, Tramacere F, Mazzolai B (2020). Taking inspiration from climbing plants: Methodologies and benchmarks—a review. Bioinspir. Biomim..

[34] Fukano Y, Yamawo A (2015). Self-discrimination in the tendrils of the vine *Cayratia japonica* is mediated by physiological connection. Proc. R. Soc. B Biol. Sci.

[35] Fukano Y (2017). Vine tendrils use contact chemoreception to avoid conspecific leaves. Proc. R. Soc. B Biol. Sci..

[36] Bowling AJ, Vaughn KC (2009). Gelatinous fibers are widespread in coiling tendrils and twining vines. Am. J. Bot..

[37] Vaughn K, Bowling A (2011). Biology and physiology of vines. Hortic. Rev. (Am. Soc. Hortic. Sci.).

[38] Gerbode SJ, Puzey JR, McCormick AG, Mahadevan L (2012). How the cucumber tendril coils. Science.

