# Supplementary material for: A study on diameter-dependent support selection of the tendrils of Cayratia japonica
Source: Sci Rep. 2022 Mar 15;12:4461. doi: 10.1038/s41598-022-08314-w (PMC8924264; doi:10.1038/s41598-022-08314-w)
Supplement: Supplementary file 1 — Supplementary Information 1. [file 41598_2022_8314_MOESM1_ESM.pdf]

Supplementary Information for

A study on diameter-dependent support selection of the tendrils of *Cayratia japonica*

Kazuya Saito[1*]

[1]Kyushu University, Faculty of Design, Fukuoka, 815-8540, Japan

[*]k-saito@design.kyushu-u.ac.jp

This PDF file includes:

Supplementary table S1, S2
Figure S1
Legends for Video 1 to 5

Other supplementary materials for this manuscript include the following:
Video S1 to S4

Table S1.
The effects of tendril length and support diameter on the coiling success of tendrils of *Cayratia japonica* (generalized linear model analysis).

Response variables	Explanatory variables	Deviance	d.f	*P* - value
Coiling success	Tendril length	12.56	1	< 0.001
	Support diameter	69.567	1	< 0.001
	Tendril length × support diameter	0.172	1	0.6786436

Table S2.
The effects of tendril length and support diameter on the first, second and third phases in coiling response of *Cayratia japonica* (generalized linear model analysis).

Response variables	Explanatory variables	Deviance	d.f	*P* - value
First coiling phase	Tendril length	0.343	1	0.558
	Support diameter	73.65	1	< 0.001
	Tendril length × support diameter	0.034	1	0.854
Second coiling phase	Tendril length	21.6736	1	< 0.001
	Support diameter	4.4496	1	0.035
	Tendril length × support diameter	3.669	1	0.055
Third phase	Tendril length	10.979	1	< 0.001
	Support diameter	24.186	1	< 0.001
	Tendril length × support diameter	5.0605	1	0.024

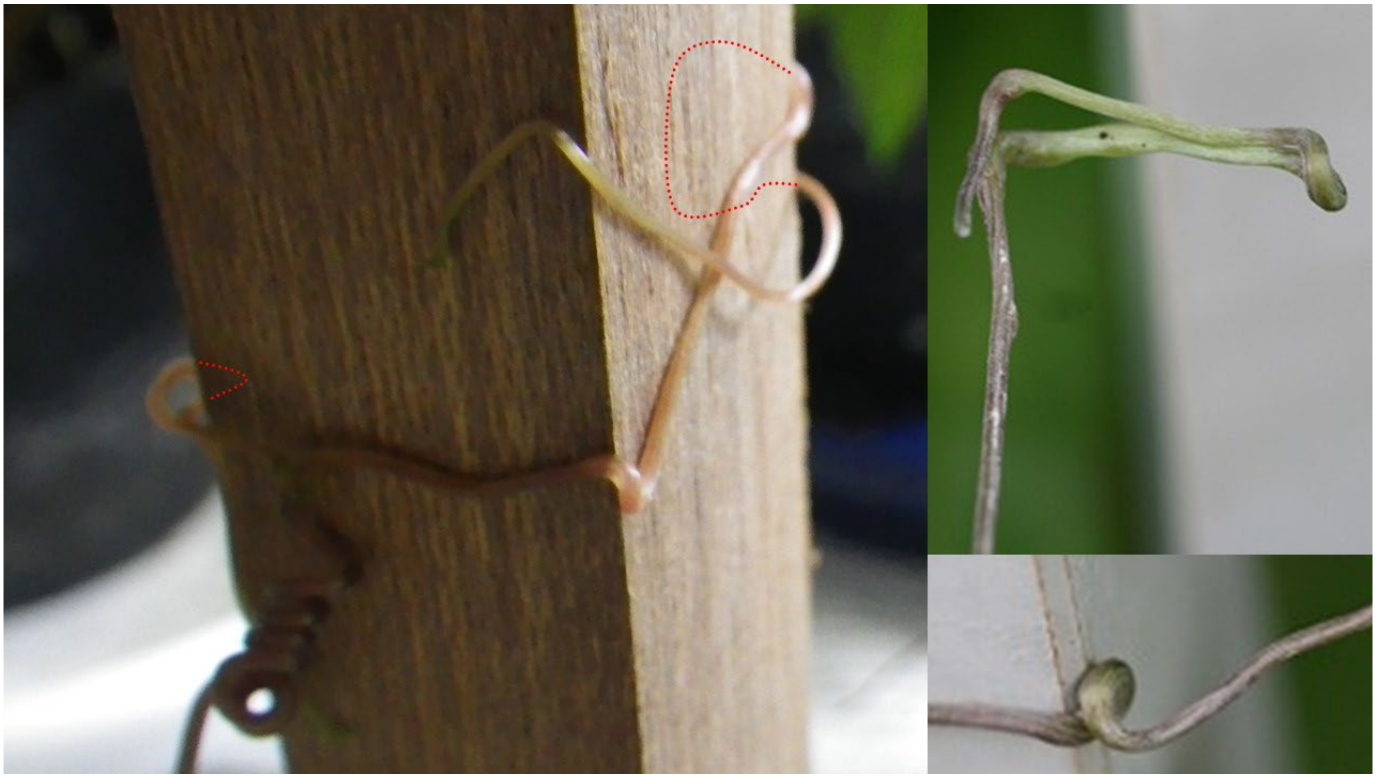

Figure S1 Examples of non-coiling grasp patterns found in a tendril of *Cayratia japonica*. Left: with a square rod. Upper light: An example of the tendril shape after grasping a square rod. Bottom light: Example of fixing to a corner.

Video Captions

Video 1
Continuous coiling found in a tendril of *Cayratia japonica*.

Video 2
Moving contact point and clip shape coiling found in a tendril of *Cayratia japonica*.

Video 3
Tip contact found in a tendril of *Cayratia japonica*.

Video 4
3D motion tracking of the tendril coiling around thin support.

Video 5
3D motion tracking of the tendril coiling around thick support.
